# A combined model for short-term traffic flow prediction based on variational modal decomposition and deep learning

**DOI:** 10.1038/s41598-025-98496-w

**Published:** 2025-05-17

**Authors:** Chuanxiang Ren, Fangfang Fu, Changchang Yin, Li Lu, Lin Cheng

**Affiliations:** 1 College of Transportation, Shandong University of Science and Technology, Qingdao, 266590 China; 2 College of Electrical Engineering and Automation, Shandong University of Science and Technology, Qingdao, 266590 China

**Keywords:** Variational modal decomposition, Graph attention network, Time convolutional networks, Short-term traffic flow prediction, Civil engineering, Computer science

## Abstract

The emergence of Deep Learning provides an opportunity for traffic flow prediction. However, uncertainty and volatility exhibited by nonlinearity and instability of traffic flow pose challenges to Deep Learning models. Therefore, a combined prediction model, VMD-GAT-MGTCN, based on variational modal decomposition (VMD), graph attention network (GAT), and multi-gated attention time convolutional network (MGTCN) is proposed to enhance short-term traffic flow prediction accuracy. In the VMD-GAT-MGTCN, VMD decomposes traffic flow data to obtain the modal components, the GAT and MGTCN are integrated to design the spatio-temporal feature model to obtain the temporal and spatial features of traffic flow. The predicted value of traffic flow modal components by spatio-temporal feature model are stacked to obtain the ultimate traffic flow prediction results. The simulation experiments with the compared models and the baseline models show that the VMD-GAT-MGTCN have superior prediction accuracy and effect. It also verifies the enhancement effect of the VMD algorithm on the prediction performance of the VMD-GAT-MGTCN and the good prediction results obtained by the VMD-GAT-MGTCN in the traffic flow mutation region.

## Introduction

In order to alleviate traffic congestion, intelligent transportation system (ITS) is widely used^1–3^. Short-term traffic flow prediction is an important part of ITS. By accurately predicting traffic flow, the congested road sections can be identified in advance, which helps the traffic managers take timely measures to regulate traffic^4^. Furthermore, based on the predicted traffic flow, the optimization of signal timing and phase control can alleviate traffic congestion and maximize road traffic efficiency^5^. In addition, traffic flow prediction can also help travelers plan travel routes in advance, guide vehicles around congested areas, and reduce travel pressure^6^.

In order to accurately predict traffic flow, scholars have proposed many methods, which include statistical-based prediction methods^7–9^, machine learning-based prediction methods^10–12^, and deep learning-based prediction methods. Among these methods, deep learning-based methods can learn the laws and features hidden inside the sample data and are extensively employed. For example, Yang et al.^13^ propose an improved long short-term memory network (LSTM) to predict traffic flow through multi-scale feature enhancement and attention mechanisms. Zhang et al.^14^ proposed a traffic flow prediction model using prediction error as fitness value and genetic algorithm to optimize the hyperparameters of time convolutional networks (TCN).

With the deepening of the research, scholars found that only considering the time characteristics is not enough for improving the traffic flow prediction accuracy, so spatial characteristics were gradually introduced to improve the accuracy and reliability of the prediction. For example, Wang et al.^15^ proposed a hierarchical traffic flow prediction protocol based on spatial-temporal graph convolutional network to achieve more accurate traffic flow prediction, which integrates the spatial and temporal dependencies of intersections, and effectively predicts the traffic flow of intersections without historical data. Gao et al.^16^ proposed a spatio-temporal traffic flow prediction model based on graph attention network (GAT) and bidirectional gated recurrent unit (BiGRU) to obtain the spatial characteristics of road network and traffic flow data, respectively.

Meanwhile, traffic flow data has the characteristics of nonlinearity and instability, which is manifested as volatility. This presents a challenge to deep learning-based prediction methods and significantly influences the prediction results^17^. The signal decomposition algorithm can decompose the traffic flow time series data into several more stable intrinsic mode functions (IMFs) to diminish data volatility. This enables the prediction model to more accurately capture the temporal features of the traffic flow data. Therefore, signal decomposition algorithms are gradually applied to traffic flow prediction^18–21^.

There are a variety of signal decomposition algorithms, such as EMD^22^, EEMD^23^, CEEMD^24^, and VMD^25^. Compared with the other three algorithms, VMD can overcome the problems of modal aliasing and end-point effect, and has strong adaptive and robustness^26^. However, the spatio-temporal features of the traffic flow exhibit a certain degree of complexity, there is still margin for research on fusing signal decomposition algorithms and deep learning methods for traffic flow prediction. Therefore, considering the spatio-temporal features and the instability of traffic flow, this paper proposes a traffic flow prediction model based on VMD and deep learning methods. The main contributions of this study include the following three aspects:

(1) A model for acquiring spatio-temporal features of traffic flow, GAT-MGTCN, is proposed, which consists of the spatial feature acquisition module (GAT) and the time feature acquisition module (MGTCN). Among them, MGTCN is designed through gated linear unit (GLU), gated tanh unit (GTU), and TCN.

(2) Based on GAT-MGTCN and VMD, a combined traffic flow prediction model is proposed, i.e., VMD-GAT-MGTCN. The model firstly decomposes the traffic flow data by VMD to obtain the traffic flow IMFs, and then IMFs are input GAT-MGTCN for traffic flow prediction, and the prediction results are obtained after stacks.

(3) Compared with the compared models based on different signal decomposition algorithms and baseline models found that the VMD-GAT-MGTCN effectively reduces the nonlinearity and instability of traffic flow data and has better prediction accuracy, especially in the traffic flow mutation region also obtains better prediction effect.

The subsequent sections of the paper are organized as follows: Sect. 2 provides an overview of the research status on short-term traffic flow prediction. Section 3 introduces the traffic flow decomposition method and the traffic flow spatio-temporal features model and establishes the VMD-GAT-MGTCN short-term traffic flow combination prediction model. Section 4 designs the experiments to validate the VMD-GAT-MGTCN and analyzes the results. Finally, in Sect. 5, this paper is summarized, and future work is suggested.

## Literature review

Regarding the prediction of short-term traffic flow, statistical-based methods are firstly used. Emami et al.^27^ proposed a Kalman filtering algorithm based on vehicle networking data for short-term traffic flow prediction on urban arterial roads. Yi et al.^28^ developed a Kalman filter prediction model with multiple linear regressions to predict the traffic congestion time. Moshe et al.^29^ studied 20, 40, and 60- second occupancy and volume collected at two different locations during peak hours through Box-Jenkins time series analysis and evaluated several autoregressive integrated moving average (ARIMA) models. Mai T et al.^30^ presented an additive seasonal vector ARIMA model to predict the short-term traffic flow. And the efficiency of the proposed prediction model was evaluated by real-time traffic flow observations. Shahriari et al.^31^ proposed E-ARIMA model combining bootstrap with ARIMA model and used ARIMA and LSTM as a compared model to verify the E-ARIMA model. Chen et al.^32^ explored spatiotemporal mobilities of highway traffic flows and presented a multiple slope-based linear regression method to predict road travel time. Although statistical-based prediction methods offer advantages such as simple structure and rapid training, it requires high-quality training data and exhibit relatively low prediction accuracy. With the current traffic environment becoming more and more complex, statistical-based prediction methods have been difficult to meet the needs.

With the advent of machine learning, machine learning-based prediction methods have been used in traffic flow prediction. Xu et al.^33^ presented a kernel K-nearest neighbor (KNN) algorithm to predict road traffic states, which maps multi-dimensional and multi-granularity road traffic state data series to high dimensions using a constructed kernel function. Wang et al.^34^ propose a KNN prediction algorithm with asymmetric losses and overcome the limitations of traditional KNN by reconstructing Euclidean distance. Wang et al.^35^ developed a regression framework with automatic parameter tuning for short-term traffic flow prediction, which used support vector machine (SVR) and Bayesian optimization for regression model and parameter selection, respectively. Hu et al.^36^ used SVR to build the traffic flow prediction model, in which model parameters are optimized by particle swarm algorithm. Su et al.^37^ introduced an incremental SVR-based short-term traffic flow prediction model that updates the prediction function in real-time through incremental learning techniques. Zhu et al.^38^ proposed a linear conditional Gaussian Bayesian network model to predict traffic flow, which considers both spatio-temporal and speed information in the traffic flow. Sun et al.^39^ Wang et al. proposed a fully automatic dynamic process KNN method for traffic flow prediction, which can automatically adjust KNN parameters with good robustness. Ryu et al.^40^ developed a short-term traffic flow prediction method that uses mutual information to construct traffic state vectors and processed the traffic state vectors using the KNN model. Lin et al.^41^ combined the SVR and KNN for short-term traffic flow prediction. The above-cited machine learning-based traffic flow prediction methods can obtain excellent prediction accuracy. However, the inability to characterize complex nonlinearity spatio-temporal correlations of traffic flow largely restricts the applicability of such prediction models.

Deep learning methods with the advantages of strong robustness and good fault tolerance have been used more and more frequently in the traffic flow prediction. Kipf et al.^42^ proposed graph convolutional network (GCN) for traffic flow prediction, which solves the shortcomings of convolutional neural networks (CNN) that can’t deal with topological maps. Koesdwiady et al.^43^ took weather as influencing factor, and designed the deep belief networks model to predict traffic flow. Sun et al.^44^ proposed a short-term traffic flow prediction model based on an improved GRU and added a bidirectional feedback mechanism to extract spatio-temporal features to further improve prediction accuracy. Tian et al.^45^ presented an approach based on LSTM to predict traffic flow with missing data, which used multi-layered temporal smoothing to deduce missing data. Yang et al.^13^ enhanced the LSTM model by introducing the attention mechanism to capture key traffic flow values, which are closely associated with the current time step. Li et al.^46^ developed a GCN model, which improves the accuracy of medium and long-term traffic prediction through adaptive mechanism and multi-sensor data correlation convolution block.

With the growing complexity of traffic flow prediction and the increasing number of factors to be considered, single deep learning prediction models mentioned above struggle to meet prediction requirements, and the combined deep learning prediction models have become a major research direction of traffic flow prediction. Chen et al.^47^ proposed a method based on dynamic space-time graph- based CNN to predict the traffic flow. In the method, the time characteristics are extracted by the spatiotemporal convolution layer based on graph, and the dynamic graph structure is predicted by the graph prediction flow. Qiao et al.^48^ proposed a combination prediction model for the short-term traffic flow using the LSTM and 1DCNN and made use of the resulting spatio-temporal characteristics for regression prediction. Sun et al.^49^ proposed a selective stacking GRU model for road network traffic volume prediction, which uses linear regression coefficient for spatial pattern mining and stacked gated cycle units for multi-road traffic prediction. Peng et al.^50^ presented a long-term traffic flow prediction method, in which dynamic traffic flow probability graph is used to model traffic network, GCN is used to learn spatial features, and LSTM unit is used to learn temporal features. Hu et al.^51^ propose an intelligent dynamic spatiotemporal graph convolution system for traffic flow prediction to improve prediction performance by capturing complex and dynamic spatial and temporal dependencies. Yu et al.^52^ proposed a traffic flow prediction method, which employed principal component analysis to analyze intersection correlation, and used convolutionally GRU and bidirectional gated recurrent units (BiGRU) to extract spatio-temporal characteristics of traffic flow. Liu et al.^53^ proposed a self-attention-based model combining CNN, LSTM, and attention mechanism to predict the short-term traffic flow. Wang et al.^54^ considered the impact of weather on the traffic flow and proposed a combined prediction model which incorporates attention mechanism, 1DCNN, and LSTM. Zhang et al.^55^ presented a combined traffic flow prediction model, which used an improved graph convolution recurrent network based on residual connected blocks and LSTM to automatically extract spatio-temporal features of traffic flow.

Although deep learning-based prediction methods have further improved the prediction accuracy, there is still the problem of not being able to comprehensively extract the features contained of the traffic flow data. The signal decomposition algorithm provides a way to solve this problem, which can decompose the original signal (data) into multiple IMFs, making it easier to fit the complex nonlinear relationships, thus improving the accuracy of data prediction. Among the various signal decomposition algorithms, VMD is used to traffic flow prediction because of its ability to overcome the problems of mode aliasing and end-point effect. Liu et al.^56^ proposed a combined prediction model for traffic flow prediction, which includes VMD, the group method of data handling neural network, BiLSTM network, and ELMAN network. And the imperialist competitive algorithm is used to optimize the combined prediction model. Zhao et al.^57^ proposed a combined traffic flow prediction model based on VMD and improved dung beetle optimization-long short term memory network. Jing et al.^58^ introduced a model based on short-term memory network to predict traffic flow, which employs VMD to tackle modal aliasing and enhance the prediction precision. The above-mentioned traffic flow prediction methods utilizing VMD achieved better prediction results. However, these methods primarily focus on the temporal features of traffic flow and ignore the influence of spatial features on the accuracy of prediction. In this paper, the spatio-temporal features of the traffic flow are considered, and the VMD, as well as GCN and TCN are used to design a combined model for the short-term traffic flow prediction.

## Methodology

### Traffic flow prediction model

The real traffic flow is nonlinear, unstable, and highly dependent on time and space, which requires the prediction model to overcome this difficulty and fully explore its time and space characteristics. VMD can decompose the traffic flow data and make its time features easy to obtain as well as reduce the instability in the data.

TCN is a novel architecture based on CNN, which not only has the characteristics of parallelism and causality, but also has a flexible receptive field, and is suitable for processing time series data such as traffic flow. Based on TCN and the gating mechanism, the traffic flow time feature acquisition module is designed, namely multi-gated time convolution network (MGTCN). GAT is obtained by introducing the attention mechanism into GCN. GCN can capture complex patterns and nonlinear relationships in the graph structure data. The attention mechanism enables the GAT to capture the complex spatial relationships among nodes, accurately express the dependency and the degree of influence between nodes by learning the attention weights and extract the nonlinear relationships between nodes. And GAT is used for the acquisition of traffic flow spatial features.

**Fig. 1:**
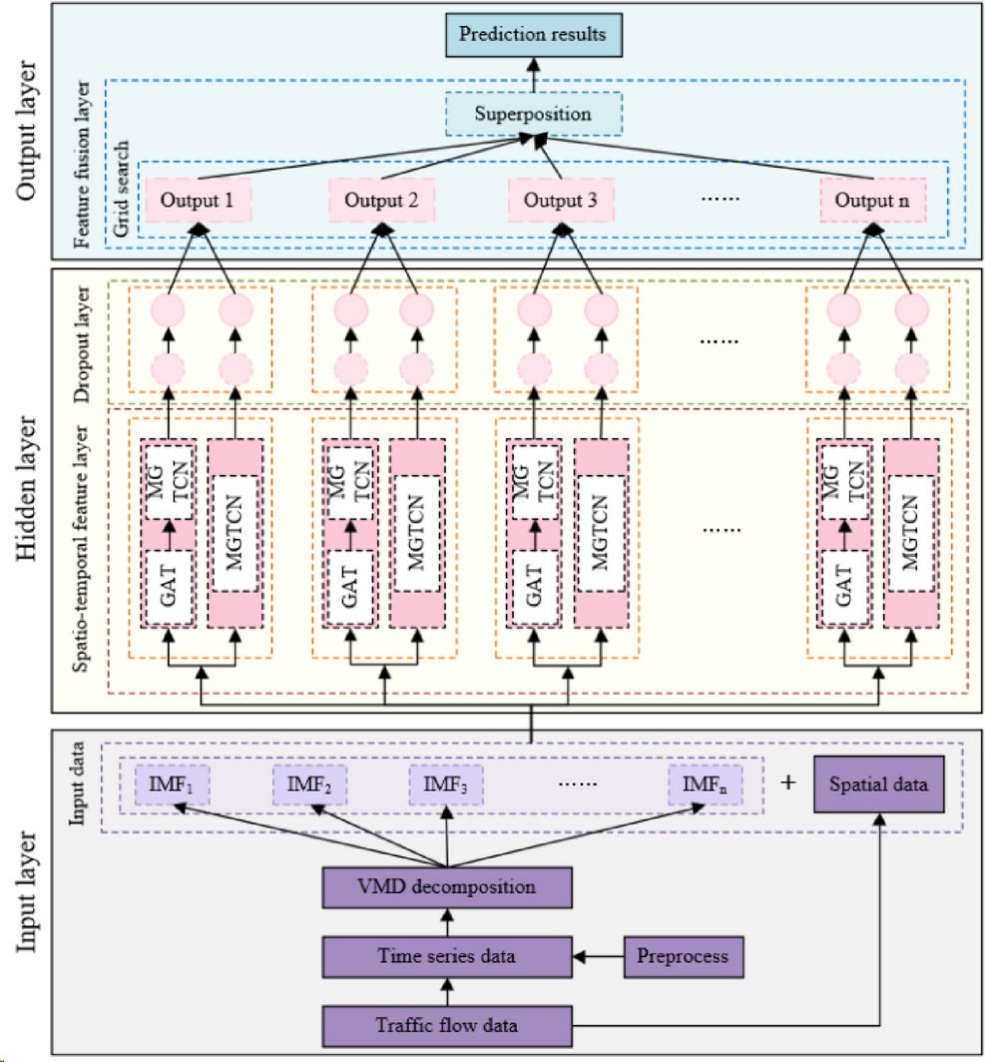
Overall framework of the VMD-GAT-MGTCN.

Based on the MGTCN and GAT, the traffic flow spatio-temporal feature model is designed and named GAT-MGTCN. On the basis of the VMD and the GAT-MGTCN, a combined traffic flow prediction model is proposed and named VMD-GAT-MGTCN. In the model, VMD is used to decompose traffic flow data and obtain the IMFs in the data, while the GAT-MGTCN extracts temporal and spatial features. From the perspective of layers, VMD-GAT-MGTCN consists of input layer, hidden layer, and output layer. The overall framework is shown in Fig. 1.

The input layer provides input data. The obtained raw traffic flow data includes the time series data of traffic flow and the spatial data of each node. After preprocessing, including data cleaning, normalization, etc., the time series data of traffic flow is obtained, and then is decomposed by VMD to obtain IMFs. Finally, the IMFs are combined with spatial data to form the input data.

The hidden layer includes the spatio-temporal feature layer and the Dropout layer. The spatio-temporal feature layer is composed of the GAT-MGTCN. GAT receives IMFs and spatial data from the input layer, constructs an adjacency matrix of the fully connected graph to represent the dependency between nodes, and forms a feature matrix with IMFs. The sliding window method is used to convert the data into a time series samples, and the feature dimension of each sample is the sum of the number of modal components and spatial features. The time series samples were input into MGTCN for time feature extraction, and a set of prediction data was obtained. At the same time, only IMFs were input into MGTCN to extract the time feature, and another set of prediction data was obtained. The two sets of prediction data are processed through the Dropout layer to prevent overfitting of the model.

Output layer includes feature fusion layer and prediction results. Feature fusion layer assigns appropriate weights to the outputs of the Dropout layer through a grid search algorithm and performs superposition. Finally, the superimposed results as predicted results are output.

### Traffic flow decomposition method

VMD possesses the characteristics of adaptive and completely non-recursive, which allows for setting the number of IMFs based on the actual situation^59^. Compared with EMD, VMD employs a variational decomposition method that enables adaptive segmentation of each component within the frequency domain of the signal. The phenomenon of mode aliasing generated by the recursive decomposition of EMD is resolved, enhancing noise robustness and reducing end-point effect. Therefore, VMD is selected as the signal decomposition algorithm to decompose traffic flow data. The essence of VMD is to construct and solve variational decomposition problem, and the specific process is as follows.

(1) Construct variational problem. Assuming that the original signal $\:S\left(t\right)$ needs to be decomposed into *M* IMFs with finite bandwidth and center frequency, the signal decomposition problem can be turned into a constrained variational problem, i.e., the sum of the estimated bandwidths of IMFs is minimized and the sum of IMFs is equal to the original signal, and the corresponding formulars are as follows:

1 $$\left\{ \begin{gathered} \mathop {{\text{min}}}\limits_{{\left\{ {{u_{\text{m}}}} \right\}\left\{ {{\omega _{\text{m}}}} \right\}}} \left\{ {\sum\limits_{m} {\left\| {{\partial _t}\left[ {\left( {\delta \left( t \right)+\frac{m}{{\pi t}}} \right)*{u_{\text{m}}}\left( t \right)} \right]{e^{ - m{u_m}t}}} \right\|_{2}^{2}} } \right\} \hfill \\ s.t.\sum\limits_{{m=1}}^{M} {{u_{\text{m}}}} =S\left( t \right) \hfill \\ \end{gathered} \right.$$

where $\:\left\{{u}_{m}\right\}$ represent the *m*th modal component, $\:\left\{{\omega\:}_{m}\right\}$ represent the *m*th center frequency after decomposition, $\:\delta\:\left(t\right)$ is the Dirac function, and * denotes the convolution operation.

(2) Solve the variational problem. Lagrange multipliers and quadratic penalty factors are introduced in Eq. (2) to solve the optimal solution for the constrained variational problem. Equation (2) is transformed into an unconstrained variational problem, and the corresponding formular is as follows:

2 $$L\left( {\left\{ {{u_m}} \right\},\left\{ {{\omega _m}} \right\},\lambda } \right)=\alpha \sum\limits_{m} {\left\| {{\partial _t}\left[ {\left( {\delta \left( t \right)+\frac{m}{{\pi t}}} \right)*{u_m}\left( t \right)} \right]{e^{ - m{\omega _m}t}}} \right\|_{2}^{2}} +\left\| {S\left( t \right) - \sum\limits_{k} {{u_m}\left( t \right)} } \right\|_{2}^{2}+\left\langle {\lambda \left( t \right),S\left( t \right) - \sum\limits_{m} {{u_m}\left( t \right)} } \right\rangle$$

where the $\:\lambda\:\left(t\right)$ is Lagrange multipliers, *α* is quadratic penalty factors and its value represents the degree of suppression of Gaussian noise interference.

The pseudo-code of VMD is presented in Algorithm 1.

**Algorithm 1:**
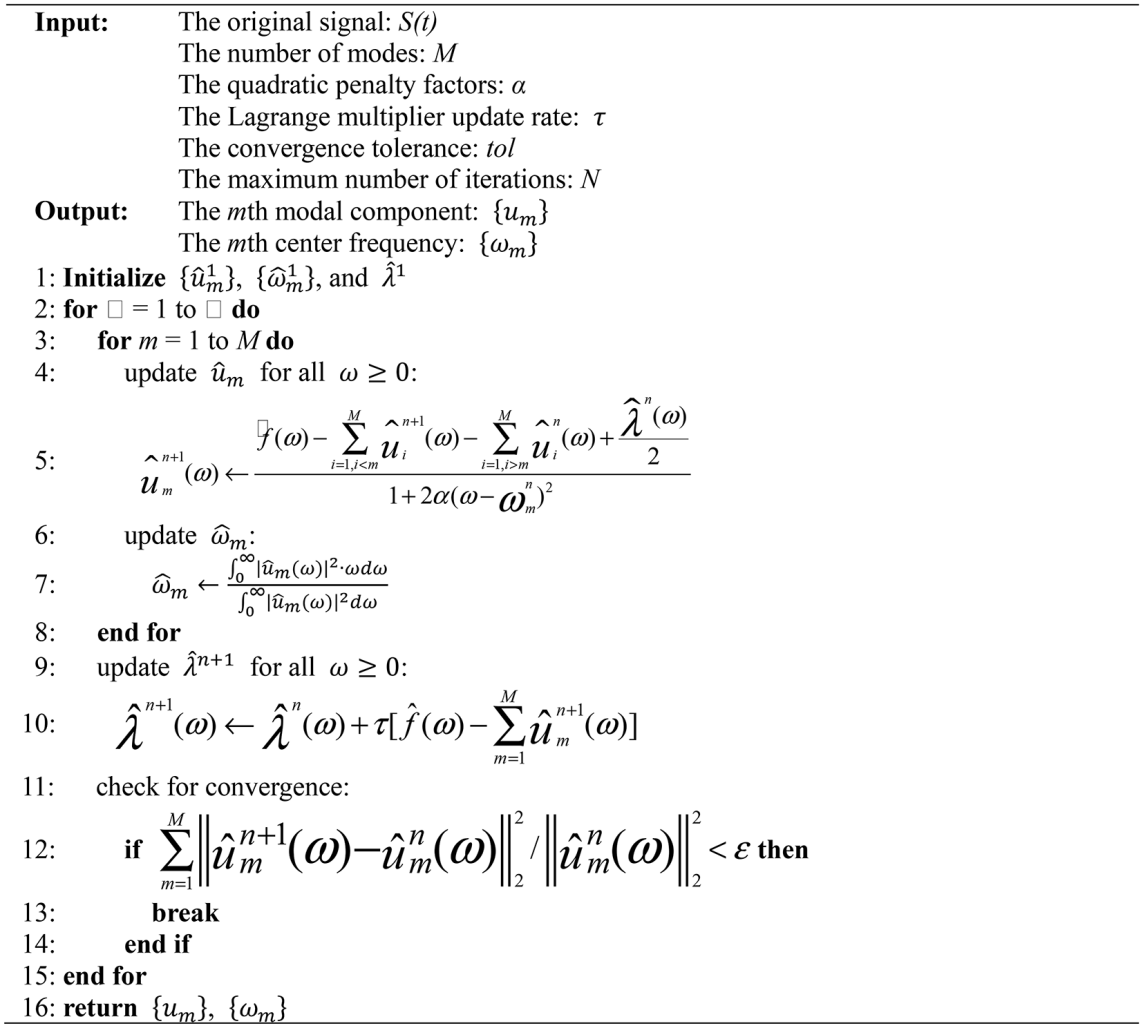
VMD.

### Traffic flow Spatio-Temporal feature model

Aiming to extract the spatio-temporal feature inherent in traffic flow data, a spatio-temporal feature model, GAT-MGTCN, is designed, which consists of spatial feature acquisition module and time feature acquisition module.

#### The structure of traffic flow Spatio-Temporal feature model

**Fig. 2:**
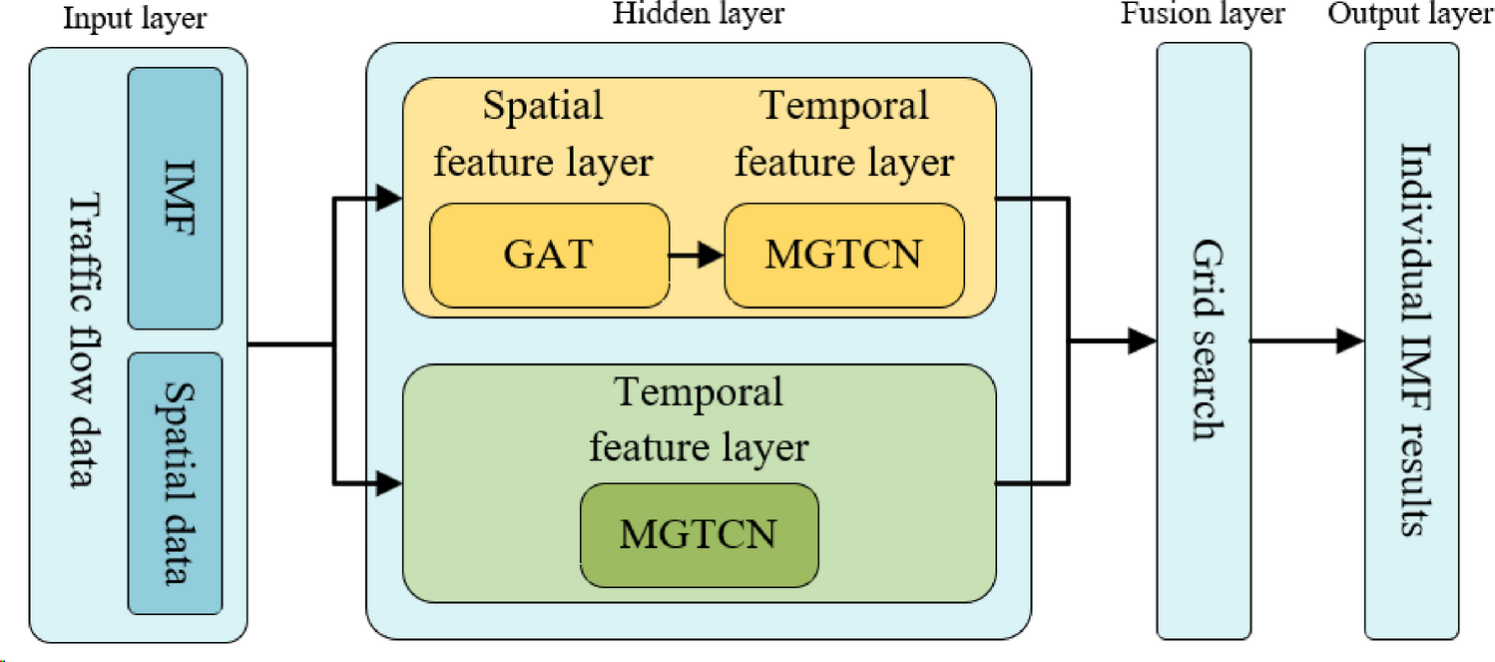
Structure diagram of the spatio- temporal feature model.

Based on the GAT and MGTCN, the GAT-MGTCN is obtained, which includes input layer, hidden layer, fusion layer, and output layer, and its structure is shown in Fig. 2.

The input layer comprises the IMFs of traffic flow data and its associated spatial data.

The hidden layer is used to extract spatio-temporal feature in traffic flow data, which consists of the spatial feature layer and temporal feature layer, namely GAT and MGTCN. Furthermore, GAT and MGTCN are combined to extract temporal and spatial features, MGTCN alone is used to extract temporal features, and then their results are fused in the feature fusion layer. This can avoid the insufficient time feature extraction and ensures deep extraction of temporal and spatial dependencies by treating spatial and temporal features separately, which together improve the overall performance of the model.

In the feature fusion layer, the grid search method is used for assigning weights to upper-layer outputs to facilitate stacking.

Finally, the output layer presents the calculation results of the spatio-temporal feature model.

#### Spatial feature acquisition module

The spatial feature acquisition module, GAT, is obtained by introducing the attention mechanism into GCN, which can dynamically learn the importance weights of neighbor nodes.

##### 1) GCN

GCN regards the graph structure as an undirected graph, firstly obtain the node information near the target node, and then weight calculation on the information to update and learn the features of the target node, so as to complete the following prediction task^60^. Furthermore, the introduction of nonlinear transformations through the activation function *ReLU* can help the GCN capture complex patterns and nonlinear relationships in the data.

The formulas for the GCN are given in Eq. (3) and Eq. (4).

3 $${\text{H}}^{1} = \sigma \left( {{\text{D}}^{{ - 1/2}} \left( {{\text{A}} + {\text{I}}} \right){\text{D}}^{{ - 1/2}} {\text{X}}\omega ^{1} } \right)$$

4 $${\text{H}}^{{\text{i}}} = \sigma \left( {{\text{D}}^{{ - 1/2}} \left( {{\text{A}} + {\text{I}}} \right){\text{D}}^{{ - 1/2}} {\text{H}}^{{{\text{i}} - 1}} \omega ^{{\text{i}}} } \right)$$

where $\:{H}^{1}$ is the feature output of the first layer of the GCN, $\:{H}^{i}$ is the feature output of *ith* layer of the GCN, $\:\sigma\:$ denotes the Sigmoid activation function, *A* denotes the adjacency matrix of the graph, *I* is the unit-diagonal matrix, *D* is the degree matrix of *A + I*, *X* denotes the feature vector of the graph, $\:{\omega\:}^{1}$ denotes the connection weights of the first layer, and $\:{\omega\:}^{i}$ denotes the connection weights of the *i*th layer.

Since the adjacency matrix *A* does not take into account its weighting, *A + I* is used as the adjacency matrix of the graph in the GCN. And in order to alleviate the problems of numerical dispersion and gradient explosion that occur during the learning process, the adjacency matrix is subjected to a normalization operation using $\:{D}^{-1/2}\left(A+I\right){D}^{-1/2}$.

##### 2) Attention mechanism

The emergence of the attention mechanism alleviates the problem of neural networks failing to obtain optimal results due to insufficient arithmetic power. The core idea of the attention mechanism is to assign a weight value to each element in the input feature. Through this mechanism, the model can pay more attention to features with significant impact on the prediction results, while reduce the attention to that having a smaller impact, and exclude irrelevant features to enhance the ability of the model to extract features^61^.

##### 3) GAT

GAT is derived from GCN and the attention mechanism. Especially, in order to steady the learning process and refine learning accuracy, GAT adopted a multi-head attention mechanism, that is, *m* independent attention mechanisms are used to calculate the hidden state of nodes, concatenate their feature, and finally obtain a new feature vector of nodes by calculating the average value of the feature. This enables the GAT to capture the complex spatial relationships among nodes, accurately express the dependency and the degree of influence between nodes by learning the attention weights, and extract the nonlinear relationships between nodes, so as to enhance GAT the acquisition of spatial features.

**Fig. 3:**
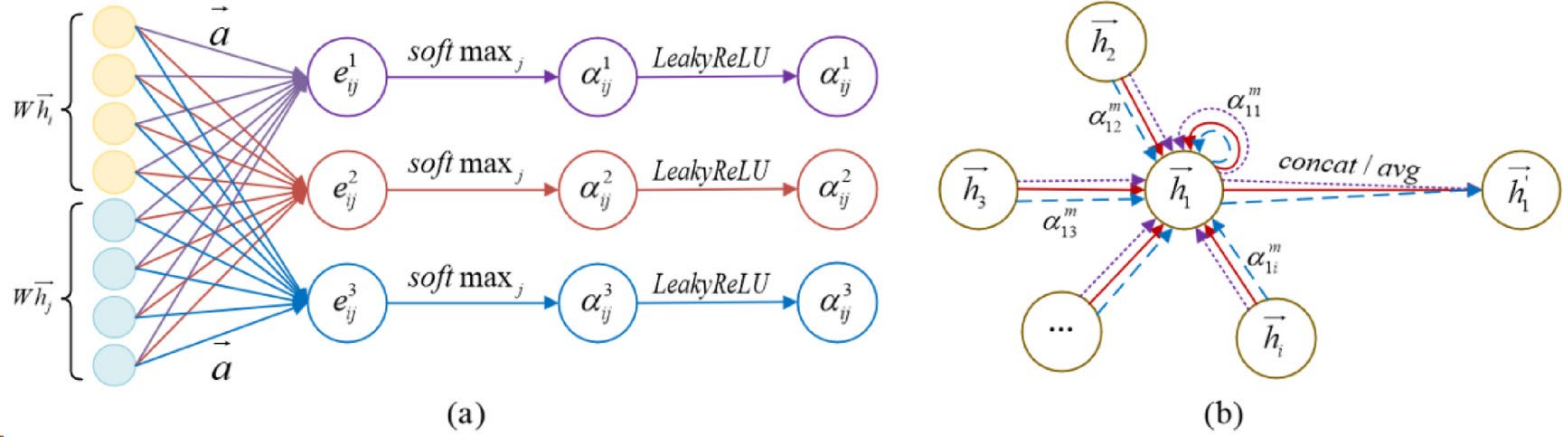
Structure diagram of graph attention layer. (**a**) Calculation of attention coefficient of multi-head, (**b**) Schematic diagram of multi-head attention mechanism with 3 heads.

In GAT, the graph attention layer is the core component and its structure diagram is shown in Fig. 3. The calculation process of the graph attention layer includes the training of weight matrix, the calculation of the attention coefficient between nodes, and the update of the feature vector.

①The training of weight matrix. The weight matrix represents the relationship between the sets of input and output node feature vectors, and the corresponding formula is as follows:

5 $$\overrightarrow {{h^{'} }} = \omega \overrightarrow {h}$$

Where $\:\omega\:$ is the weight matrix, $\overrightarrow h$ is the set of input node feature vectors, and $\overrightarrow {{h^{'} }}$ is the set of output node feature vectors.

②The calculation of the attention coefficient between nodes. The attention coefficient between nodes is calculated as follows:

6 $${\alpha _{ij}}={\text{soft}}m{\text{ax}}\left( {{{\text{x}}_j}{{\text{e}}_{ij}}} \right)=\frac{{{\text{exp}}\left( {{{\text{e}}_{ij}}} \right)}}{{\sum\limits_{{k \in {N_i}}} {\exp \left( {{e_{ik}}} \right)} }}$$

where $\:{\alpha\:}_{ij}$ denotes the attention coefficient from nodes *i* to *j*, softmax is a normalized function, $\:{e}_{ij}$ denotes the importance of nodes *j* to *i*, and *exp* is the exponential function.

In Eq. (7), the calculation formula of $\:{e}_{ij}$ is as follows:

7 $${e_{ij}}=att(\omega {\overrightarrow h _i},\omega \overrightarrow {{h_j}} )$$

Add LeakyReLU nonlinear function to Eq. (8) for activation, and the attention coefficient formula is fully expanded and can be expressed as:

8 $${\alpha _{ij}}=\frac{{\exp (Leaky\operatorname{Re} lu({a^T}[\omega {{\overrightarrow h }_i}\left\| {\omega {{\overrightarrow h }_j}]))} \right.}}{{\sum\limits_{{k \in {N_i}}} {\exp (Leaky\operatorname{Re} lu({a^T}[\omega {{\overrightarrow h }_i}\left\| {\omega {{\overrightarrow h }_k}]))} \right.} }}$$

where $\:{a}^{T}$ denotes the transpose matrix of the forward propagation weight vector matrix of the single-layer neural network, $\:{N}_{i}$ denotes the neighboring nodes of node *i*, and $[\parallel ]$denotes the function that concatenation node *i* and *j*.

③The update of feature vector. The weighted summation and average value of the features are calculated to obtain the new feature vector of node *i*, and the formular is as follows:

9 $$\overrightarrow {h_{i}^{\prime }} =\sigma \left( {\frac{1}{M}\sum\limits_{{m=1}}^{M} {\sum\limits_{{j \in {N_i}}} {\alpha _{{ij}}^{m}{\omega ^m}\overrightarrow {{h_j}} } } } \right)$$

where $\:\sigma\:$ is the Sigmoid function, $\:{\alpha\:}_{ij}^{m}$ is the normalized attention coefficient of the *m*th head attention mechanism, and $\:{\omega\:}^{m}$ is the weight matrix of the corresponding linear transformation.

The pseudo-code of GAT is presented in Algorithm 2.

**Algorithm 2:**
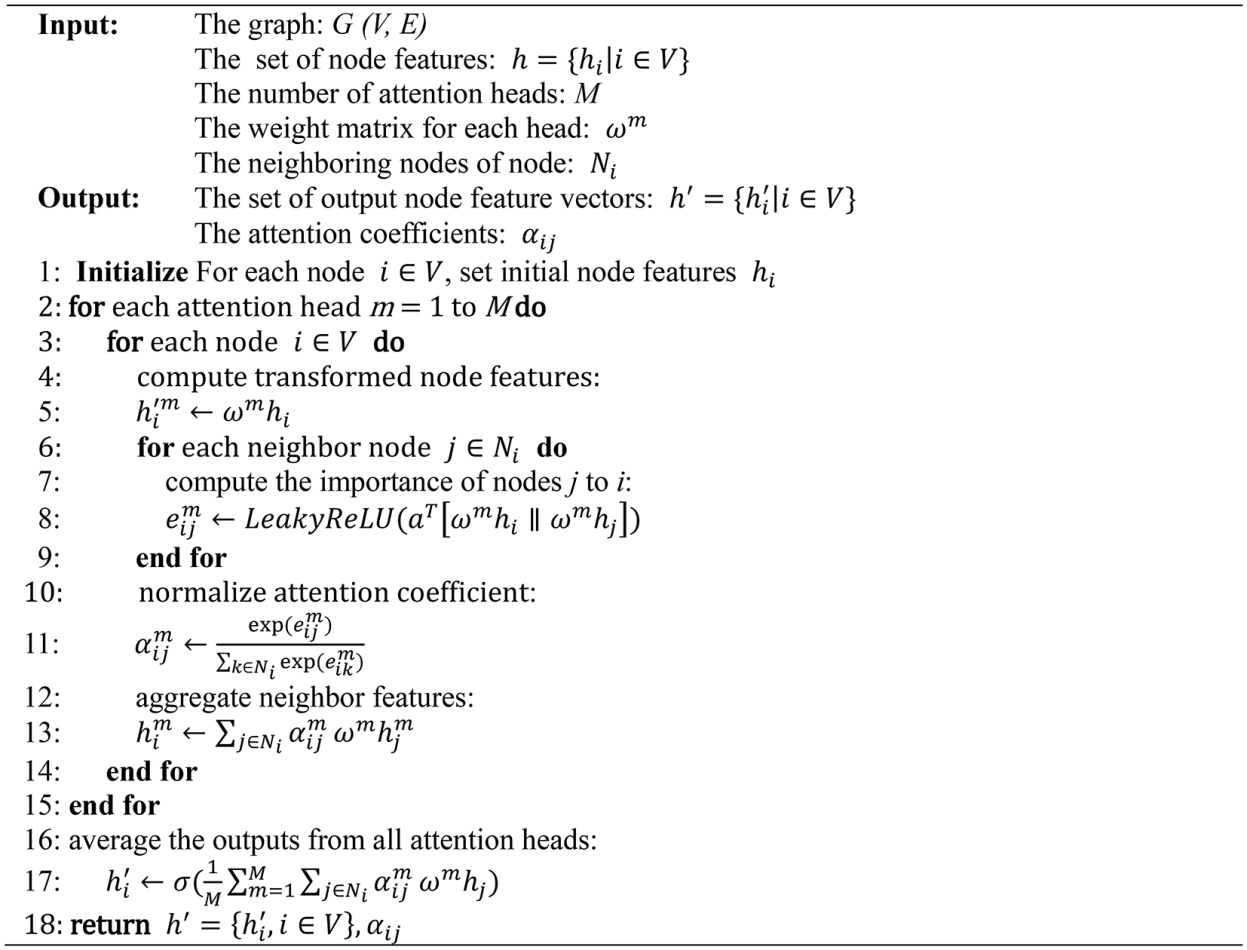
GAT.

#### Time feature acquisition module

Based on TCN and the gating mechanism, the traffic flow time feature acquisition module is designed, namely MGTCN.

**Fig. 4:**
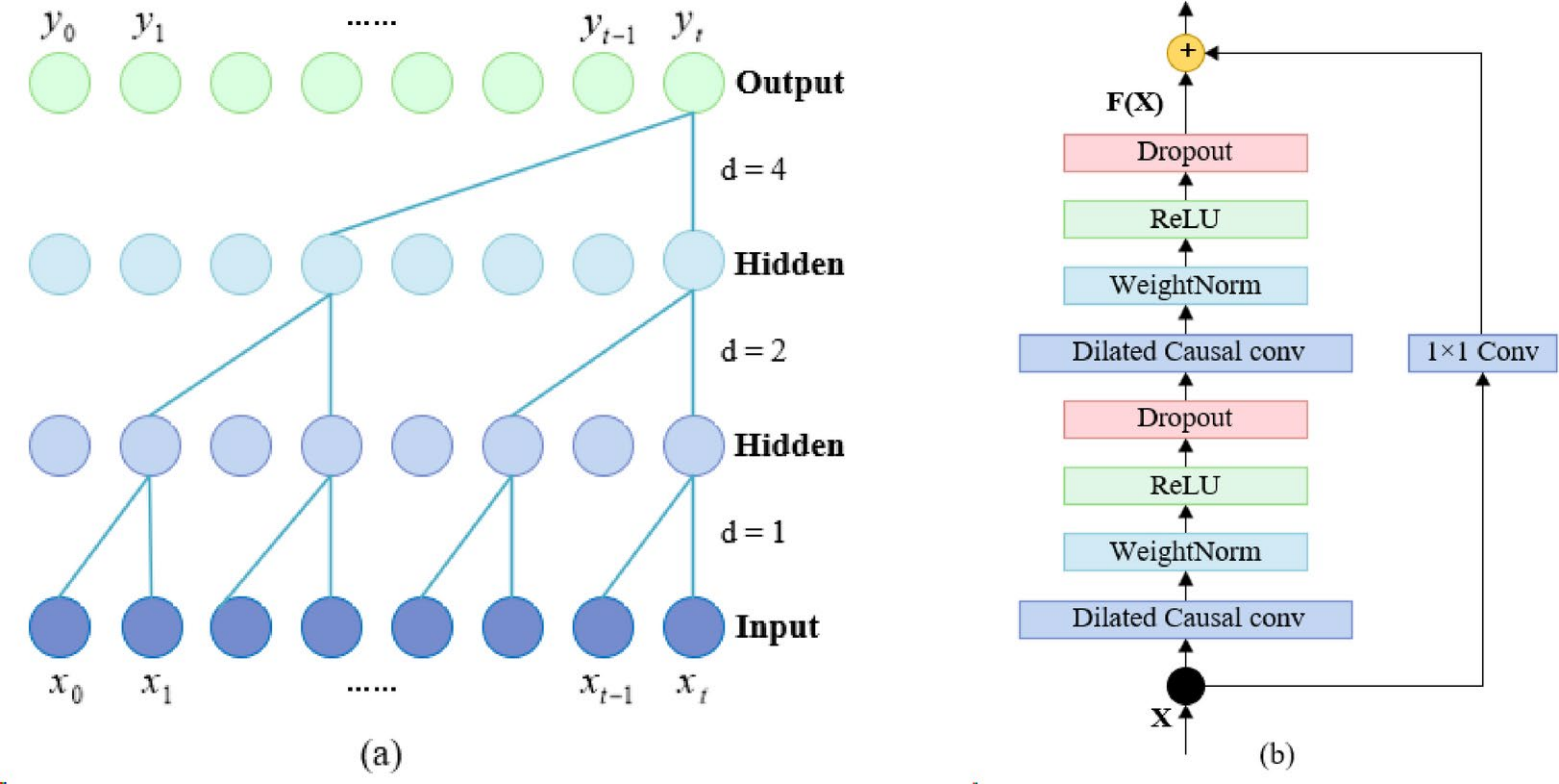
Structure diagram of TCN. (**a**) Dilated causal convolution network, (**b**) TCN residual block.

*1)* TCN: TCN is a novel architecture based on CNN and consists of dilated causal convolution network and residual blocks^62^. The structure diagram of TCN is shown in Fig. 4. Specifically, Fig. 4 (a) demonstrates the dilated causal convolution network with two hidden layers, dilation factors d is 1, 2, 4, and filter size is 2. In Fig. 4(b), a 1 × 1 convolution is introduced to the residual path to ensure a consistent number of features. Dilated causal convolution networks with fewer layers provide TCN with a larger receptive field. The residual block can learn the constant mapping function, so that the neural network can transfer information across the layers and avoid gradient vanishing and explosion. Furthermore, TCN, which not only has the characteristics of parallelism and causality, but also has a flexible receptive field, is suitable for processing time series data such as traffic flow.

In dilated causal convolution network, for a one-dimensional sequence input $\:X\in\:{R}^{n}$ and a filter $\:f:\left\{0,\:\:\dots\:\:,\:\:k-1\right\}$, the dilated convolution operation is calculated as follows:

10 $$F(s)=X * f=\sum\limits_{{l=0}}^{{k - 1}} {f(l){X_{s - d \times l}}}$$

Where *s* is element of the sequence, *F(s)* denotes the result of dilated convolution operation, *X* denotes the input data, *f* denotes the filter for the convolution operation, *d* denotes the dilation rate, and *l* is the number of layers in which the convolution kernel is located.

Residual blocks include dilated causal convolution network, weight normalization, *ReLU* activation functions, and Dropout. Dilated causal convolution network expands the receptive field by introducing dilation into the convolution kernel while preserves causality, which helps to capture long-term dependencies in time series. Weight normalization improves training stability and generalization ability. *ReLU* introduces nonlinear characteristics to enable the network to learn complex nonlinear relationships. Dropout helps prevent overfitting and makes the network more robust.

The residual block adds the original input *X* of the model to the constant mapping function *F(X)* to obtain the final output *P*, which is calculated as follows:

11 $$P{\text{ }}={\text{ ReLU }}\left( {X{\text{ }}+{\text{ }}F\left( X \right)} \right)$$

##### 2) MGTCN

**Fig. 5:**
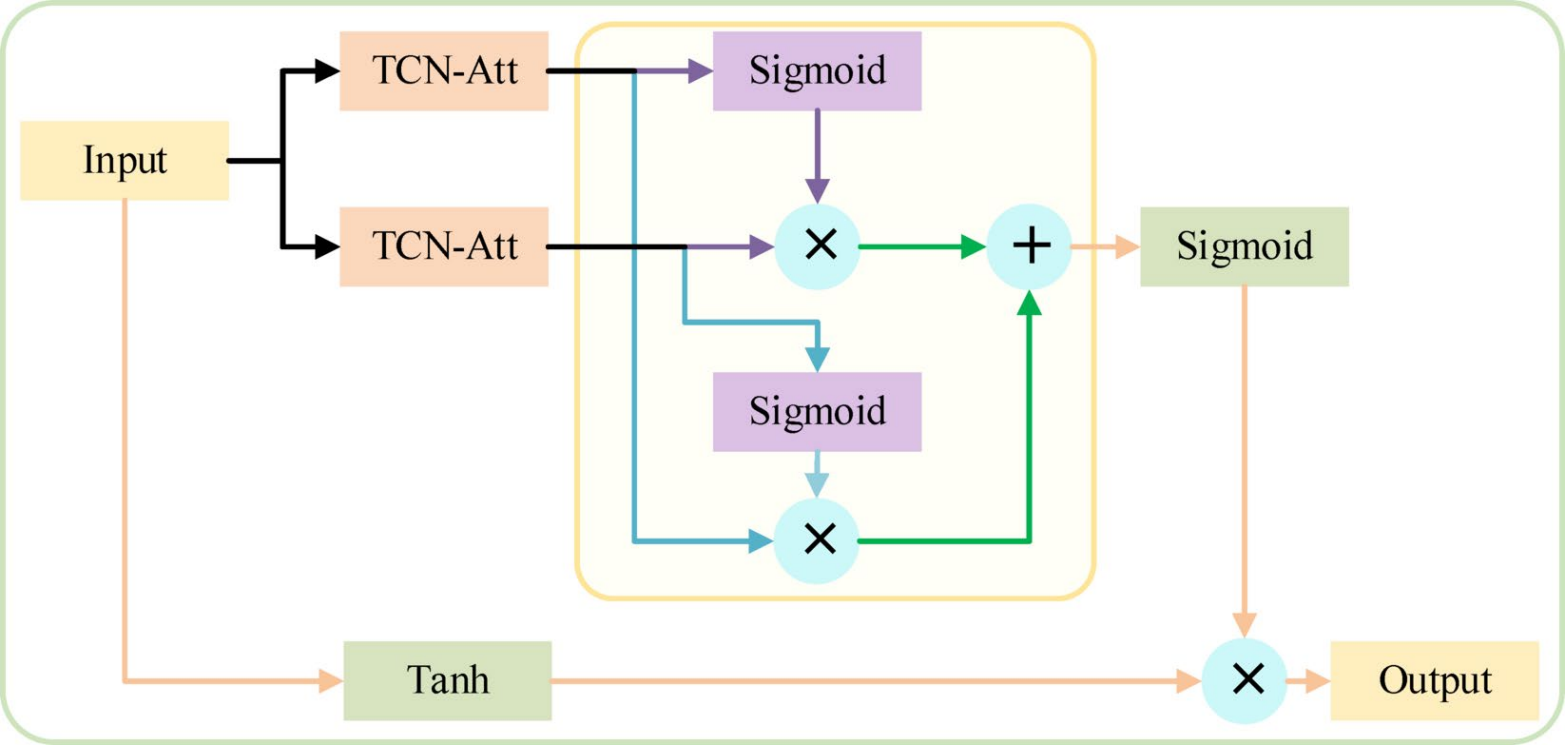
Structure diagram of MGTCN.

Adding gating mechanisms to neural networks can control the flow of information and features, thereby improving the convergence speed and computational efficiency of the networks^63^. Based on this, MGTCN is designed, which consists of two TCNs with the attention mechanism (TCN-ATT), two GLU gating mechanisms, and one GTU gating mechanism. And the structure diagram of MGTCN is shown in Fig. 5. The MGTCN can extract and fuse temporal features at different time scales through multiple gating mechanisms, which improves the capability to capture temporal features.

In MGTCN, attention mechanism enhances the ability of TCN to acquire temporal features of traffic flow. The processing of double GLU gating mechanisms for the results of two TCNs with attention mechanism can increase nonlinear features and filter useless messages. Then the two processed results are stacked by assigning weights through the attention mechanism. Finally, the stacked results and the original data are processed through the GTU gating mechanism to determine the features to be retained or discarded, and the final output results are obtained. The formula for the MGTCN is as follows:

12 $$h=\tanh {\text{ }}\left( {{\omega _{\text{1}}} * {{\text{X}}_1}} \right) \cdot \sigma \left( {{\omega _{\text{2}}} * {{\text{X}}_2}} \right)$$

where *h* is the output, **∙** denotes the multiplication operation of the matrix, * denotes the convolution operation, $\:{\omega\:}_{1}$ and $\:{\omega\:}_{2}$ are the parameters that need to be learned, $\:\sigma\:$ is *Sigmoid* function used to filter useless information, $\:{X}_{1}$ denotes the input data, and $\:{X}_{2}$ is the output obtained by double GLU gating.

And $\:{X}_{2}$ can be calculated as follows:

13 $${\text{X}}_{2} = \omega _{7} \left( {\left( {\omega _{3} * {\text{X}}_{3} } \right) \cdot \sigma \left( {\omega _{4} * {\text{X}}_{4} } \right)} \right) \cdot \omega _{8} \left( {\sigma \left( {\omega _{5} * {\text{X}}_{3} } \right) \cdot \left( {\omega _{6} * {\text{X}}_{4} } \right)} \right)$$

where $\:{\omega\:}_{3}$, $\:{\omega\:}_{4}$, $\:{\omega\:}_{5}$, $\:{\omega\:}_{6}$, $\:{\omega\:}_{7}$, and $\:{\omega\:}_{8}$ are the model parameters to be learned, $\:{X}_{3}$ denotes the output result of TCN-Att1, and $\:{X}_{4}$ is the output result of TCN-Att2.

The pseudo-code of MGTCN is presented in Algorithm 3.

**Algorithm 3:**
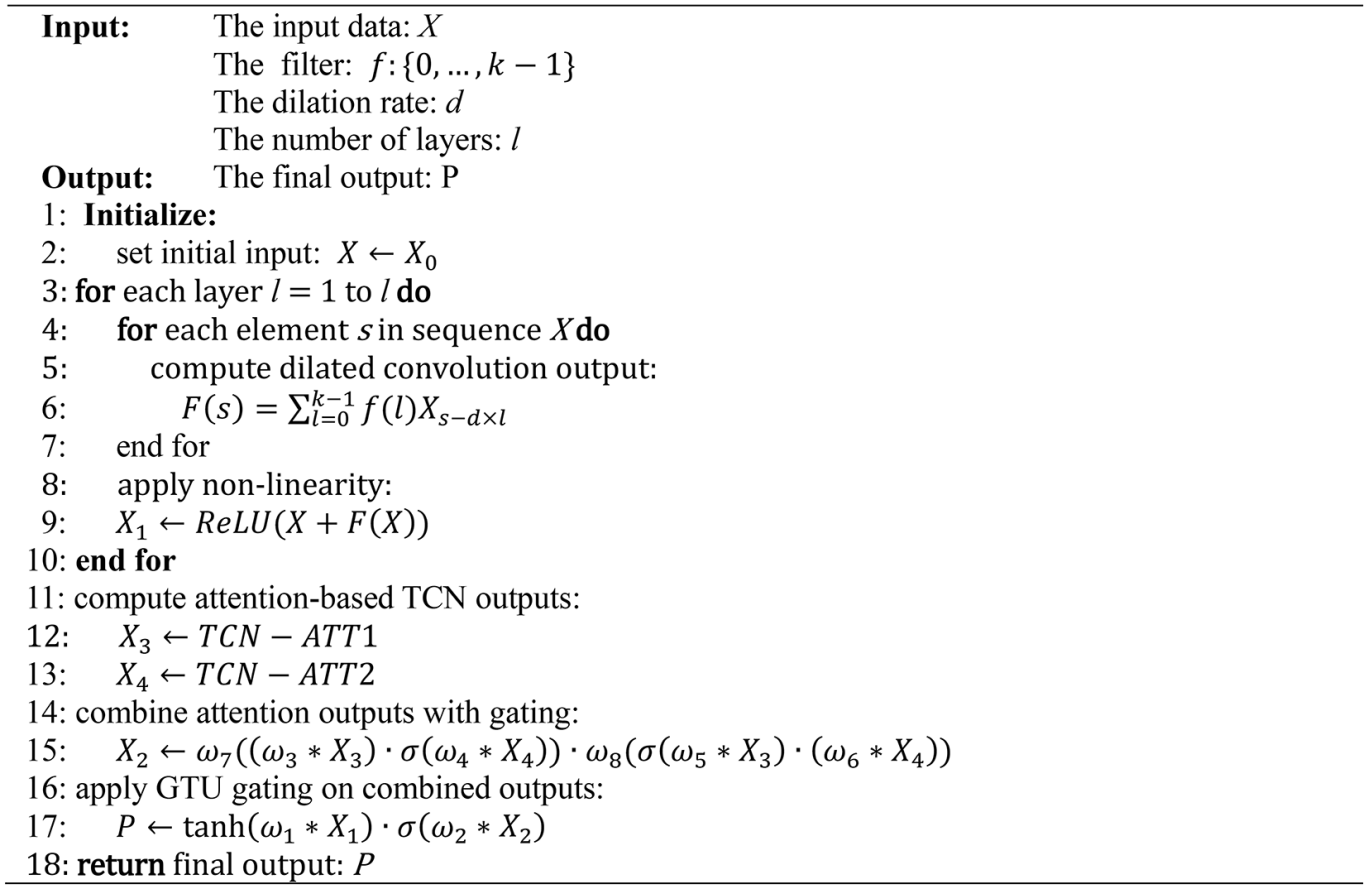
MGTCN.

## Experiments and analysis of results

### Experimental data

The publicly available dataset PeMS04 is selected for the experiments to validate the VMD-GAT- MGTCN model. The dataset contains data collected by 307 sensors on the freeway system in the Los Angeles, California area at five-minute intervals. In PeMS04, the traffic flow data of 14 consecutive days from January 1 to January 14, 2018 are selected as the experimental dataset after processing for redundant, abnormal, and missing data, where the first 12 days’ data constitute the training set, while the remaining 2 days’ data form the test set. In addition, to ensure that the selection of data points is unbiased, the traffic flow prediction for data collection point 109 was randomly chosen as an example for experimental analysis, serving to evaluate the model’s prediction performance.

### Experimental environment and model evaluation index

The hardware and software conditions of the experimental environment are provided in Table 1.

**Table 1:** Experimental environment.

Software and hardware configuration	Configuration parameter
CPU	CPU Intel i5-13500 H@2.60 GHz
RAM	16G
Programming language	Python 3.8.8
Deep learning framework	PyTorch 1.10.2

The mean absolute error (MAE), the root mean square error (RMSE), the mean absolute percentage error (MAPE), and the coefficient of determination (R^2^) are selected as the evaluation indexes. MAE reflects the error between the predicted value and the actual value, RMSE reflects the deviation of the predicted value from the actual value, MAPE is the error after eliminating the influence of proportional effect, and R^2^ reflects the correlation of linear strength between the predicted value and the actual value. The calculation formulas of the three evaluation indexes are as follows:

14 $$MAE=\frac{1}{M}\sum\limits_{{m=1}}^{M} {\left| {{Y_T} - {Y_D}} \right|}$$

15 $$RMSE=\sqrt {\frac{1}{M}\sum\limits_{{m=1}}^{M} {{{\left( {{Y_T} - {Y_D}} \right)}^2}} }$$

16 $$MAPE=\frac{{\text{1}}}{{\text{M}}}\sum\limits_{{{\text{m}}=1}}^{{\text{M}}} {\left| {\frac{{{{\text{Y}}_{\text{T}}} - {{\text{Y}}_{\text{D}}}}}{{{{\text{Y}}_{\text{T}}}}}} \right|}$$

17 $$\mathop R\nolimits^{2} = 1 - \frac{{\sum\limits_{{m = 1}}^{M} {\mathop {(\mathop Y\nolimits_{D} - \mathop Y\nolimits_{T} )}\nolimits^{2} } }}{{\sum\limits_{{m = 1}}^{M} {\mathop {(\mathop Y\nolimits_{D} - \overline{{\mathop Y\nolimits_{D} }} )}\nolimits^{2} } }}$$

where *M* is the total number of samples, $\:{Y}_{T}$ is the predicted value, $\:{Y}_{D}$ is the actual value of the sample, and $\:\stackrel{-}{{Y}_{D}}$ is the mean of the actual values.

### Model training and experimental results

(1) Model Parameter Settings.

#### 1) VMD parameter settings

In VMD, the penalty factor is set to 2000, the noise tolerance is 0.1, and the uniform initialization coefficient is 1. In addition, there is a crucial parameter in VMD, i.e. the number of signal decomposition. If it is too large, modal aliasing may occur, meaning that modes of different frequencies are insufficiently separated, resulting in overlap in the frequency domain. If it is too small, the complexity of the signal may not be fully captured, leading to an incomplete representation of the original signal and the loss of critical information in the reconstructed signal. To determine its optimal value, all other parameters were held constant, and the numbers of IMFs varied from a lower value of 3 and incrementally increasing to 10. Experiments were conducted at different numbers of IMFs, and the results are presented in Table 2.

**Table 2:** Decomposition results for different numbers of IMFs.

IMFs	Number							
	3	4	5	6	7	8	9	10
IMF1	0.0321	0.0276	0.0285	0.0263	0.0261	0.0260	0.0261	0.0250
IMF2	0.0370	0.0343	0.0324	0.0326	0.0319	0.0297	0.0271	0.0197
IMF3	0.3453	0.0873	0.1364	0.0339	0.0299	0.0365	0.0367	0.0402
IMF4		0.1812	0.2600	0.1001	0.0973	0.0636	0.0540	0.0406
IMF5			0.3188	0.1811	0.1697	0.1652	0.1347	0.0876
IMF6				0.3564	0.2439	0.2959	0.2012	0.1301
IMF7					0.3628	0.3977	0.3100	0.1768
IMF8						0.4608	0.3998	0.2584
IMF9							0.4656	0.3582
IMF10								0.4660

As can be seen in Table 2, with the increase in the number of IMFs, the center frequency of the last IMF exhibits an upward trend and starts to stabilize from a value of 8. To avoid the phenomenon of modal aliasing, the number of signal decompositions is therefore selected as 8.

#### 2) Deep Learning Hyperparameter Settings

In the GAT model, the multi-head attention mechanism is set to 3 heads, and the number of the neural units in the hidden layer is 64. For the MGTCN model, the first layer of TCN contains 128 neural units with expansion factors of [1, 2, 4], while the second hidden layer contains 64 neural units with expansion factors of [1, 2, 4]. Adam optimization algorithm is employed with an initial learning rate of 0.001. The training process involves 100 rounds, utilizing the mean square error function as the loss function, and a training batch size of 32. These parameter settings were obtained from several experiments. In the experiments, various parameter combinations were tested, and the optimal settings were determined based on the model’s performance on the test set.

#### (2) Results and analysis

**Fig. 6:**
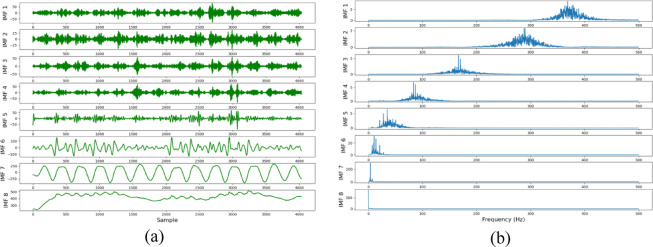
VMD decomposition results. (a)IMFs of traffic flow time series data, (b) Center frequencies of each IMF.

The traffic flow time series data are input into the VMD-GAT-MGTCN, and was decomposed firstly into 8 IMFs through VMD. The 8 IMFs and their corresponding center frequency were obtained, as shown in Fig. 6. It can be seen that each IMF clearly captures a specific frequency in the traffic flow time series data, and different IMFs are relatively independent from each other, without modal aliasing and phenomena. In addition, the various IMFs are smooth and have no obvious high-frequency components, and there is no end effect. This indicates that the VMD decomposition is effective and accurate.

**Fig. 7:**
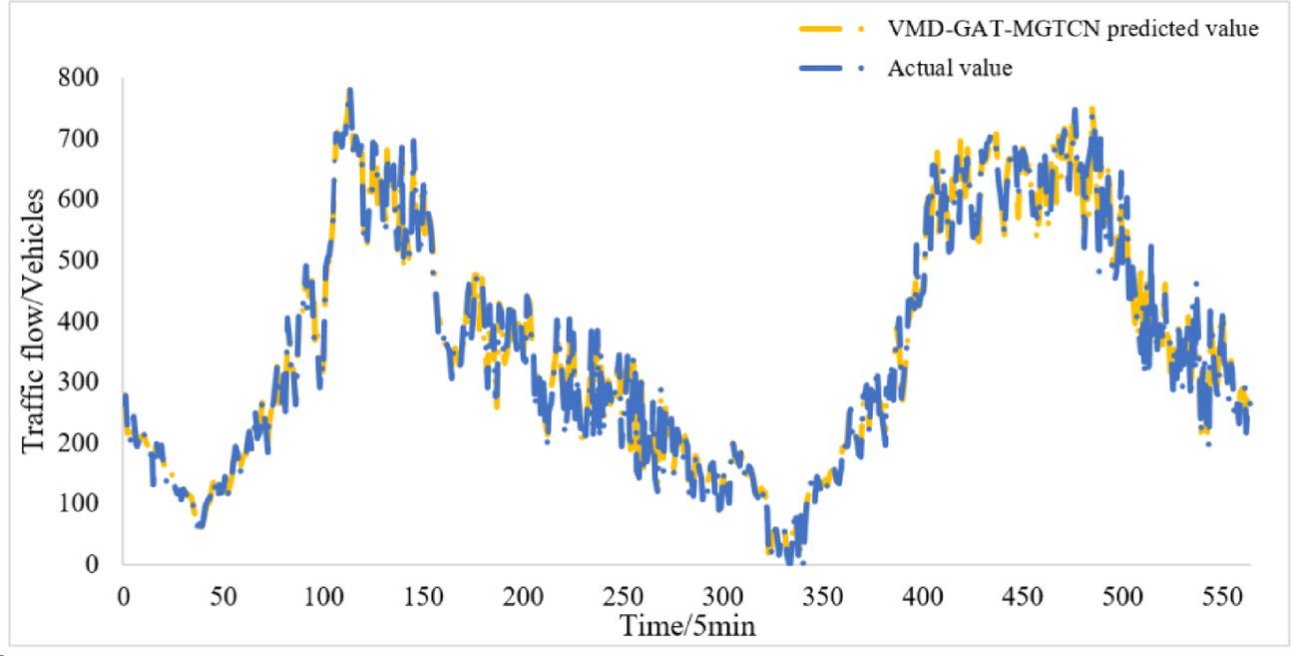
Prediction results of the VMD-GAT-MGTCN.

Then, the IMFs are integrated with the spatial data of traffic flow to form the input data. The input data is processed through hidden layer and stacked by feature fusion layer in VMD-GAT-MGTCN, and the prediction results are obtained. The prediction results on the test set can be seen in Fig. 7. Further, the error analysis of the prediction results is performed, which yields the evaluation index MAE, RMSE, MAPE, and R^2^ as presented in Table 3.

**Table 3:** Evaluation indexes value of the VMD-GAT-MGTCN.

Model	MAE	RMSE	MAPE (%)	*R* ^2^
VMD-GAT-MGTCN	15.59	21.64	11.75	0.9853

From Fig. 7, it can be found that results of the VMD-GAT- MGTCN fit the actual traffic flow remarkably well and also match the trend of the actual traffic flow. This indicates that the VMD-GAT-MGTCN employing VMD for decomposing the traffic flow data and considering the spatio-temporal features of traffic flow data and achieves a better prediction result. Furthermore, it can be found from Table II that the values of the three evaluation indexes are small, which illustrates the excellent predictive efficacy of the VMD-GAT-MGTCN.

### Signal decomposition algorithm experiment and analysis

To verify the effect of VMD on the prediction performance of the VMD-GAT-MGTCN, the spatio-temporal feature model, GAT-MGTCN, is selected as the compared model, i.e., the traffic flow data are directly input into GAT-MGTCN for traffic flow prediction without VMD decomposition. Furthermore, three types of commonly used signal decomposition algorithms EMD, EEMD, and CEEMD are selected, and based on the VMD-GAT-MGTCN, the compared models EMD-GAT-MGTCN, EEMD-GAT-MGTCN, and CEEMD-GAT-MGTCN are designed for traffic flow prediction experiments.

#### 1) Model parameter settings

The average decomposition number for signals in EMD, EEMD, and CEEMD is set to 100, and in EEMD and CEEMD, the ratio of standard deviation of additional noise to standard deviation of signal to be decomposed is 0.2. The initial learning rate, training rounds, loss function, and training batch size of the compared models are the same as the VMD-GAT-MGTCN.

#### 2) Results and analysis

**Fig. 8:**
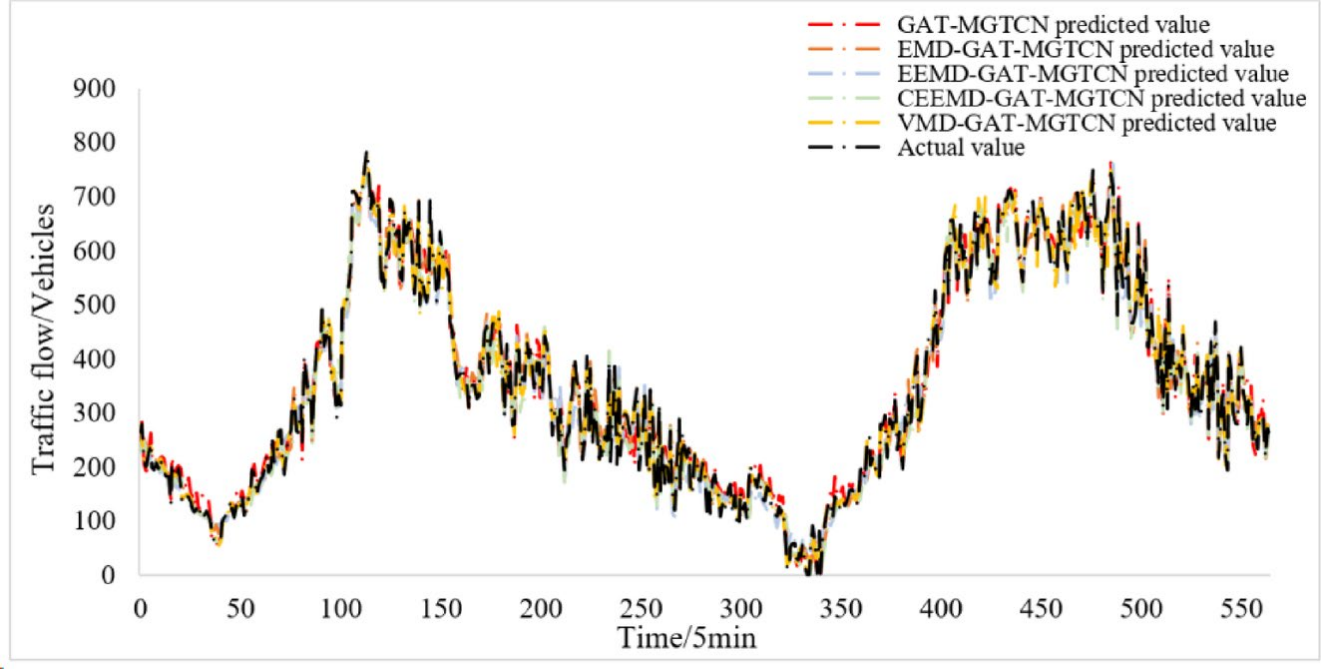
Prediction results of the compared models.

The compared models, GAT-MGTCN, EMD-GAT-MGTCN, EEMD-GAT- MGTCN, and CEEMD-GAT-MGTCN are trained and tested under the same dataset, and the prediction results for the test set are obtained, as shown in Fig. 8. In addition, to facilitate a more effective comparison between the VMD-GAT-MGTCN and other models, the prediction results of the VMD-GAT-MGTCN are also shown in Fig. 8.

**Fig. 9:**
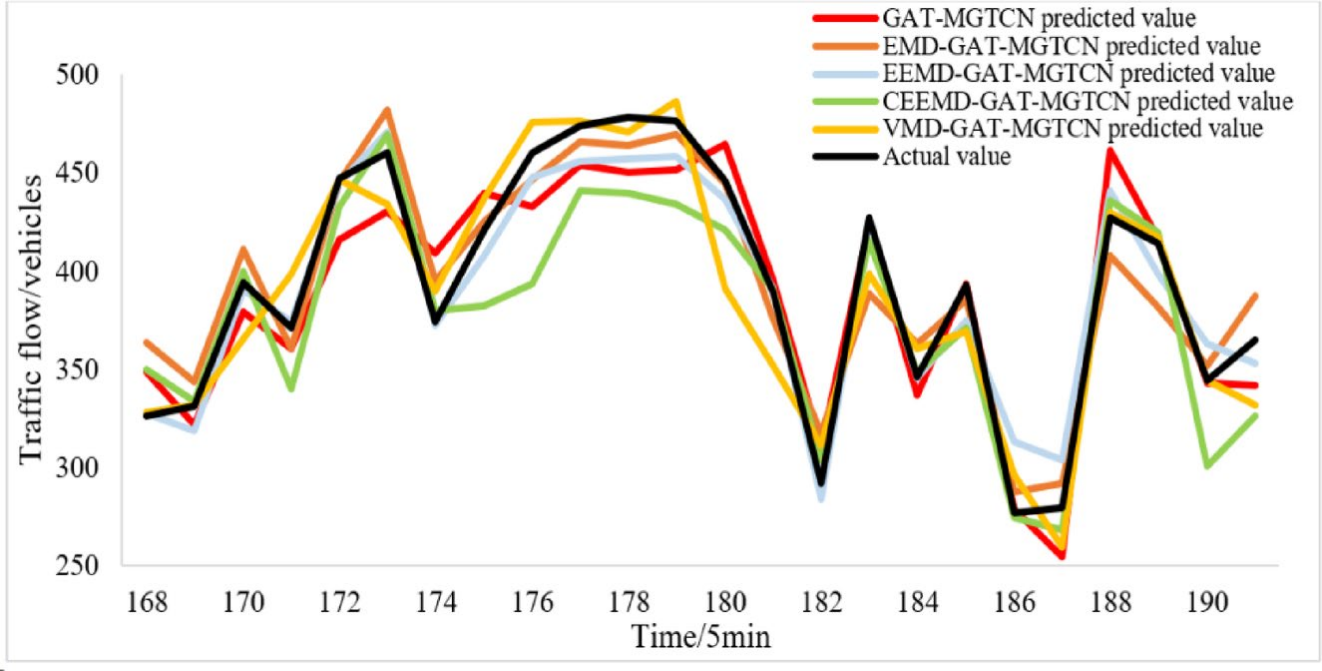
Prediction results of the compared models for two hours.

Figure 8 illustrates that both the VMD-GAT-MGTCN and the compared models maintain a high degree of overlap with the actual traffic flow values, and yield good prediction results. In order to more clearly observe the differences in the prediction results of each model, the two consecutive hours’ results are randomly selected and displayed in Fig. 9. The Fig. 9 clearly indicates that compared with GAT-MGTCN, EMD-GAT-MGTCN, EEMD-GAT- MGTCN, and CEEMD-GAT-MGTCN, the VMD-GAT-MGTCN with prediction values more fitting the actual traffic flow values obtains the better prediction results.

Furthermore, the evaluation indexes MAE, RMSE, MAPE, and R^2^ are calculated and shown in Table 4. According to Tables 3 and 4, it can be found that, compared with GAT-MGATCN, the evaluation indexes of EMD-GAT-MGATCN, EEMD-GAT-MGATCN, CEEMD-GAT-MGATCN, and VMD-GAT-MGATCN have been reduced in different degrees. This is because the traffic flow time series data before decomposition is nonlinear and prone to mutation, while after decomposition through EMD, EEMD, CEEMD and VMD, the traffic flow modal components are obtained which more stable and tend to be linear, thus enabling the prediction model to more accurately obtain the temporal features and improve the accuracy of prediction. In addition, according to Tables 3 and 4, it can also be found that all evaluation indexes of VMD-GAT-MGTCN are the smallest. This is because the VMD algorithm effectively solves mode aliasing and end effect problem in the other three signal decomposition algorithms, while reducing the instability of traffic flow data, thus resulting in improved prediction performance. Although R^2^ of EEMD-GAT-MGATCN is better than VMD-GAT-MGATCN, MAE, RMSE, and MAPE of EEMD-GAT-MGATCN are all larger than VMD-GAT-MGATCN, indicating that although EEMD-GAT-MGATCN can better capture the overall trend, the deviation of specific values is large. In general, VMD-GAT-MGATCN is better.

**Table 4:** Evaluation indexes of the compared model.

Model	MAE	RMSE	MAPE (%)	*R* ^2^
GAT-MGATCN	20.87	28.39	19.36	0.9796
EMD-GAT-MGATCN	19.87	28.2	18.31	0.9777
EEMD-GAT-MGATCN	17.05	22.25	16.1	0.9879
CEEMD-GAT-MGATCN	17.87	22.61	16.95	0.9823

### Experiments and analysis between VMD-GAT-MGTCN and baseline models

Four commonly used deep learning traffic flow prediction models, namely LSTM model, GRU model, GAT model, and BiGRU model, are selected as the baseline models.

#### (1) Baseline model parameter settings

The initial learning rate, number of training rounds, loss function, and training batch size of the baseline models are the same as those of the VMD-GAT-MGTCN, and the rest of the hyperparameter settings are shown in Table 5.

**Table 5:** Baseline models hyperparameter settings.

Model	Number of hidden neurons
LSTM	64
GRU	128
GAT	32
BiGRU	64

#### (2) Results and analysis

**Fig. 10:**
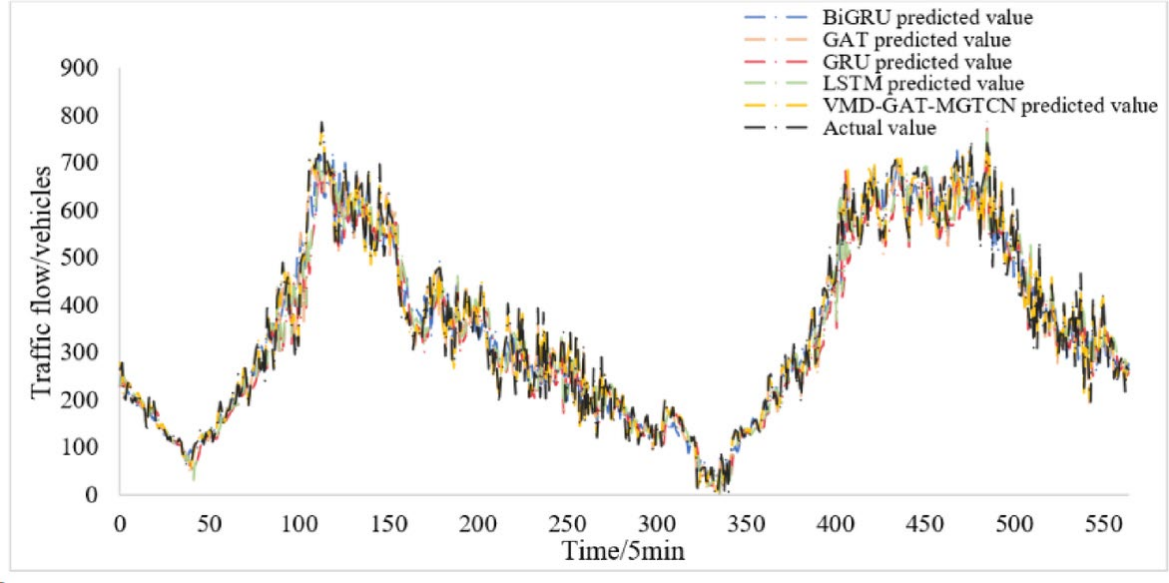
Prediction results of the baseline model.

According to the parameter settings, the baseline models LSTM, GRU, GAT, and BiGRU are trained and tested under the same dataset, and the results are obtained and displayed in Fig. 10. For comparison, the prediction results of the VMD-GAT-MGTCN are also included in Fig. 10.

**Fig. 11:**
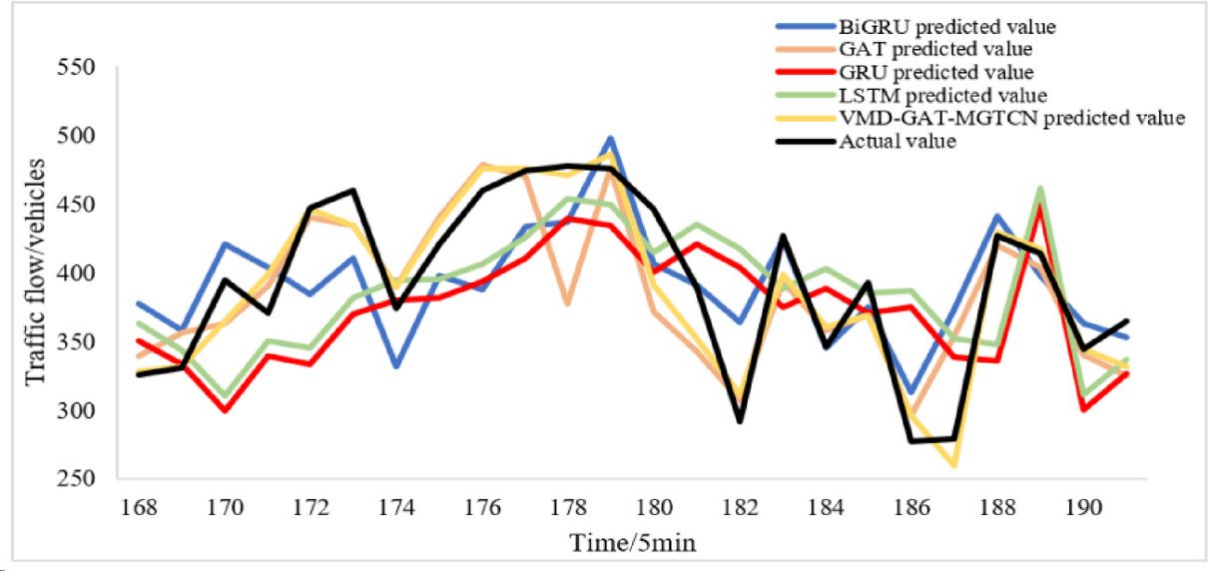
Prediction results of the baseline model for two hours.

Figure 10 illustrates that both the VMD-GAT-MGTCN and the baseline models maintain a good overlap with the actual traffic flow values, and better prediction results are achieved. In order to observe the differences in the prediction results of each model more clearly, the two consecutive hours’ results are randomly selected for two hours and are shown in Fig. 11. Based on Fig. 11, it is found that compared with the four baseline models, the VMD-GAT-MGTCN has a higher degree of overlap with the actual values of the traffic flow. This indicates the prediction results of the VMD-GAT-MGTCN are more aligned with actual traffic flow values and obtain better prediction effect.

Furthermore, the evaluation indexes MAE, RMSE, MAPE, and R^2^ are calculated and shown in Table 6. According to Tables 3 and 6, compared with the four baseline models, the VMD- GAT-MGTCN has the smallest values of the evaluation indexes. This is because the VMD-GAT-MGTCN can better acquire the nonlinearity and instability features in traffic flow data and overcome the shortcomings of the baseline models that only acquires single feature, which results in better prediction accuracy than the baseline models.

**Table 6:** Evaluation indexes of the baseline models.

Model	MAE	RMSE	MAPE (%)	*R* ^2^
BiGRU	24.55	31.6	30.66	0.9549
GAT	23.41	30.11	14.63	0.9720
GRU	36.69	41.41	33.8	0.9066
LSTM	35.6	39.98	32.7	0.9060

#### (3) Analysis of prediction results of traffic flow mutation region

Whether the traffic flow prediction method can obtain high accuracy in the traffic flow mutation region is an important reflection of prediction performance. To confirm the prediction capability of the VMD-GAT-MGTCN in the traffic flow mutation region, four traffic flow mutation regions are selected from Fig. 10, namely 90–100, 110–120, 250–260, and 485–495, which are numbered in chronological order as mutation region I, mutation region II, mutation region III, and mutation region IV.

**Fig. 12:**
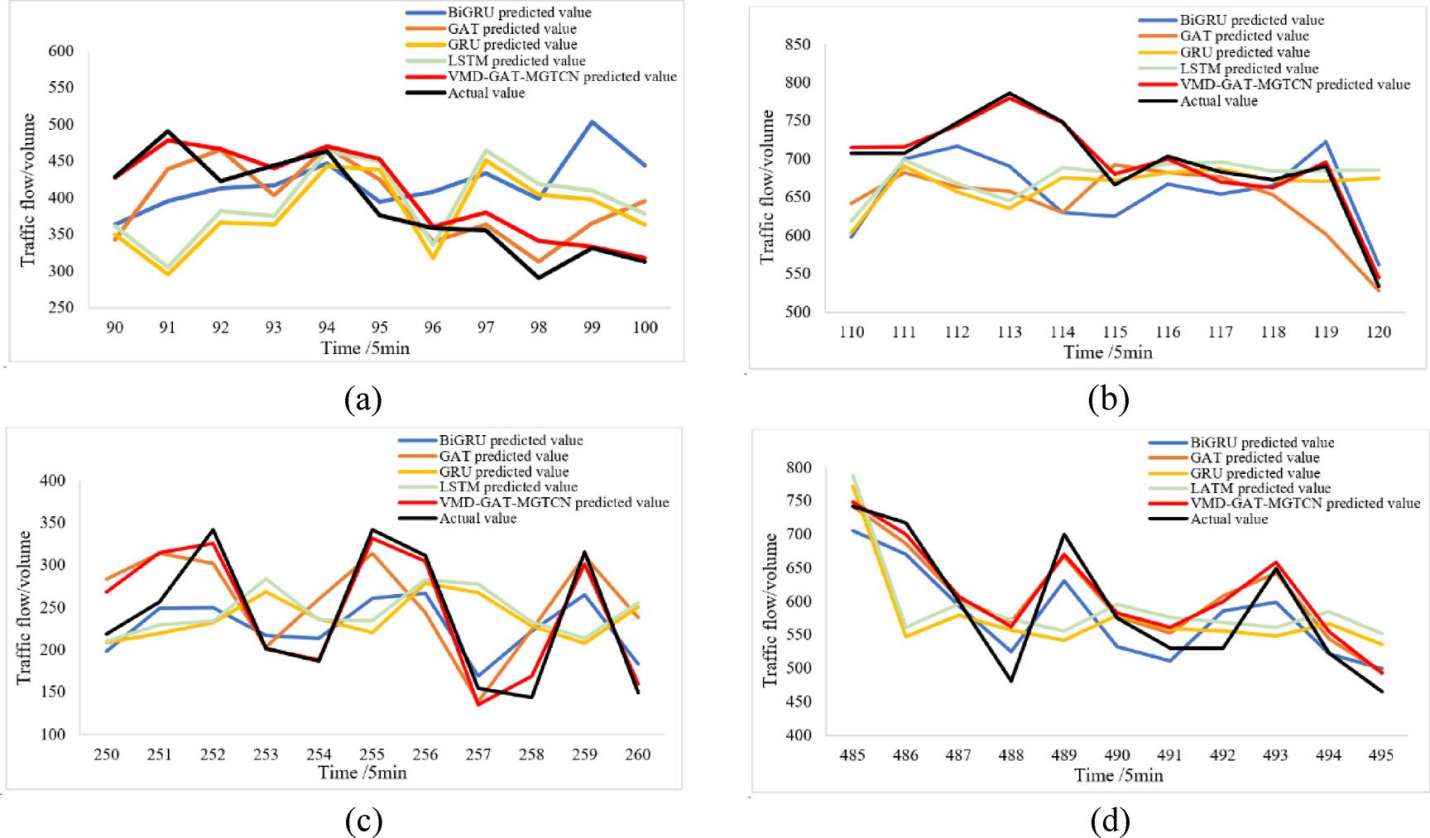
Prediction results of five models in mutation regions. (**a**) Mutation region I, (**b**) Mutation region II, (**c**) Mutation region III, (**d**) Mutation region IV.

According to the experiment, the prediction results of the VMD-GAT-MGTCN and the four baseline models in the four traffic flow mutation regions are obtained, which are illustrated in Fig. 12. Furthermore, MAE, RMSE, and MAPE are calculated and presented in Table 7.

**Table 7:** Evaluation indexes in the baseline traffic flow mutation regions.

Mutation region	Model	MAE	RMSE	MAPE (%)
Mutant region I	BiGRU	31.55	41.61	31.66
	GAT	23.91	33.19	14.70
	GRU	46.29	61.34	33.79
	LSTM	45.60	59.98	35.70
	VMD-GAT-MGTCN	15.59	23.65	11.75
Mutant region II	BiGRU	60.43	74.18	10.45
	GAT	59.60	73.22	10.17
	GRU	104.79	120.04	16.67
	LSTM	92.70	108.70	14.90
	VMD-GAT-MGTCN	5.56	6.55	0.92
Mutant region III	BiGRU	34.35	43.09	13.77
	GAT	33.88	47.54	14.58
	GRU	48.80	63.96	19.02
	LSTM	49.42	63.40	19.46
	VMD-GAT-MGTCN	12.46	15.68	5.04
Mutant region IV	BiGRU	36.94	41.87	6.22
	GAT	28.86	40.43	5.36
	GRU	66.36	85.18	10.89
	LSTM	71.14	84.63	11.96
	VMD-GAT-MGTCN	24.29	31.28	4.52

Figure 12 indicates that compared to the four baseline models, the prediction results of the VMD-GAT-MGTCN exhibit a higher degree of overlap with the actual values. And Table 7 indicates that the evaluation indexes for the VMD-GAT-MGTCN are smallest in the four mutation regions. This indicates that the prediction effect of the VMD-GAT-MGTCN outperforms the baseline models. The reason is that in the traffic flow mutation regions, the baseline models fail to predict such mutations of the traffic flow and continue to maintain the previous trend, thus showing the opposite trend to the actual value, and even if the traffic flow mutation is predicted, the error is large, resulting in a decrease of the prediction accuracy. However, the VMD-GAT-MGTCN overcomes the above drawbacks and improves the prediction accuracy in the mutation region.

### Conclusion and future work

A short-term traffic flow combined prediction model is proposed, VMD-GAT-MGTCN, which is based on VMD, GCN, attention mechanism, and TCN. The combined prediction model mainly consists of VMD and spatio-temporal feature model. The role of VMD is to decompose the traffic flow data into IMFs and reduce its instability. The spatio-temporal feature model is composed of GAT and MGTCN. The GAT is derived from adding multiple attention mechanisms in the GCN and used to capture spatial features. The MGTCN is composed of TCN and gating mechanisms and used to extract temporal features.

In order to verify the performance of the VMD-GAT-MGTCN, the PeMS04 dataset is selected, and the experiments are designed. The experimental results indicate that the VMD-GAT-MGTCN employing VMD for decomposing the traffic flow data and considering the spatio-temporal characteristics of traffic flow data achieved excellent predictive performance. Furthermore, the compared models GAT-MGTCN, EMD-GAT-MGTCN, EEMD-GAT-MGTCN, and CEEMD-GAT-MGTCN are designed and the effective function of the VMD on the prediction performance of the VMD-GAT-MGTCN is verified. In addition, compared with baseline models the VMD-GAT-MGTCN has superior prediction accuracy, and particularly in the traffic flow mutation region also obtains better prediction results.

The proposed model in this paper integrates the temporal and spatial features of traffic flow and introduces VMD to reduce the instability of traffic flow data. Simulation experiments demonstrate that good traffic flow prediction results are achieved by the proposed model. However, the actual traffic flow data often contains not only temporal and spatial features but also other factors such as cycle time, environment, and vehicle speed, etc. Therefore, considering more factors in traffic flow prediction should be further researched.

## Data Availability

The datasets generated and/or analysed during the current study are available upon request by contact with the corresponding author.
